# Supplementary material for: Light-Dependent Translation Change of Arabidopsis psbA Correlates with RNA Structure Alterations at the Translation Initiation Region
Source: Cells. 2021 Feb 4;10(2):322. doi: 10.3390/cells10020322 (PMC7914831; doi:10.3390/cells10020322)
Supplement: Supplementary file 1 [file cells-10-00322-s001.pdf]

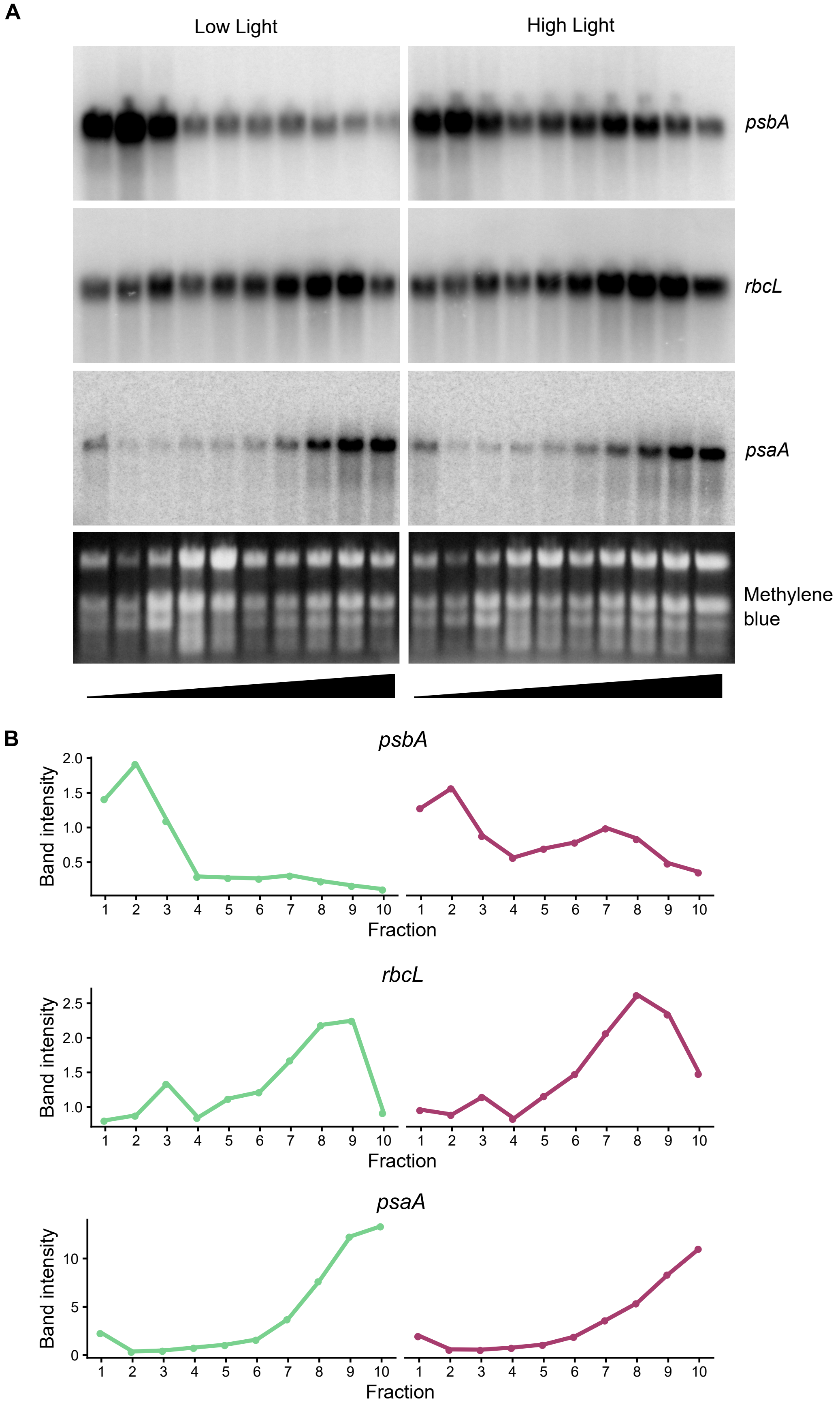

Supplemental Figure S1 Polysome analysis of *psbA* and *rbcL* translation in young plants: A, Analysis of *psbA* and *rbcL* translation in 17-18 days old plants after low light and high light treatment. *psaA* (encoding the PsaA subunit of photosystem I) is included as an example of a gene that is not upregulated in high light. Extracts from plants treated the same way as the plants analyzed by DMS-

MaPseq (Figure 1,3) were subjected to sucrose-gradient centrifugation, fractionated into ten fractions, and RNA was extracted from each. The upper panels show RNA gel blots probed for *psbA*, *rbcL*, and *psaA* mRNA, respectively. The lower panels show methylene blue-stained rRNAs. The gradients are schematically depicted as black triangles at the bottom: less dense fractions containing free mRNA are on the left, denser fractions containing polysome-associated mRNA actively engaged in translation are on the right. **B**, Quantification of the amount of *psbA*, *rbcL*, and *psaA* mRNA in each fraction normalized to the average amount of the five least dense fractions.

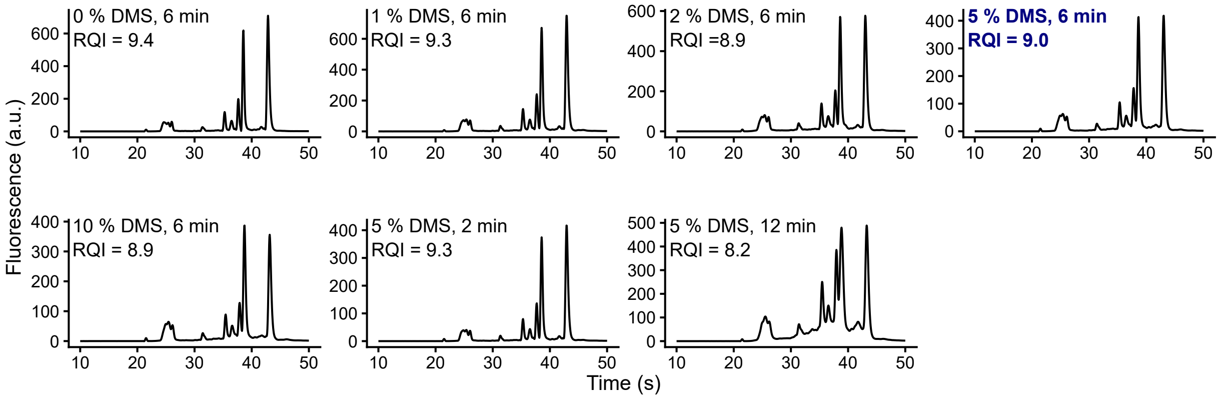

Supplemental Figure S2 RNA quality after DMS treatment: Quality assessment of RNA isolated after treatment with different DMS concentrations and different infiltration times. The results of capillary electrophoresis (Experion, Bio-Rad) are shown and the RNA Quality Index (RQI) is given (max = 10). Marked in bold and blue is the treatment that was used for RNA secondary structure probing (Figure 1 and 3).

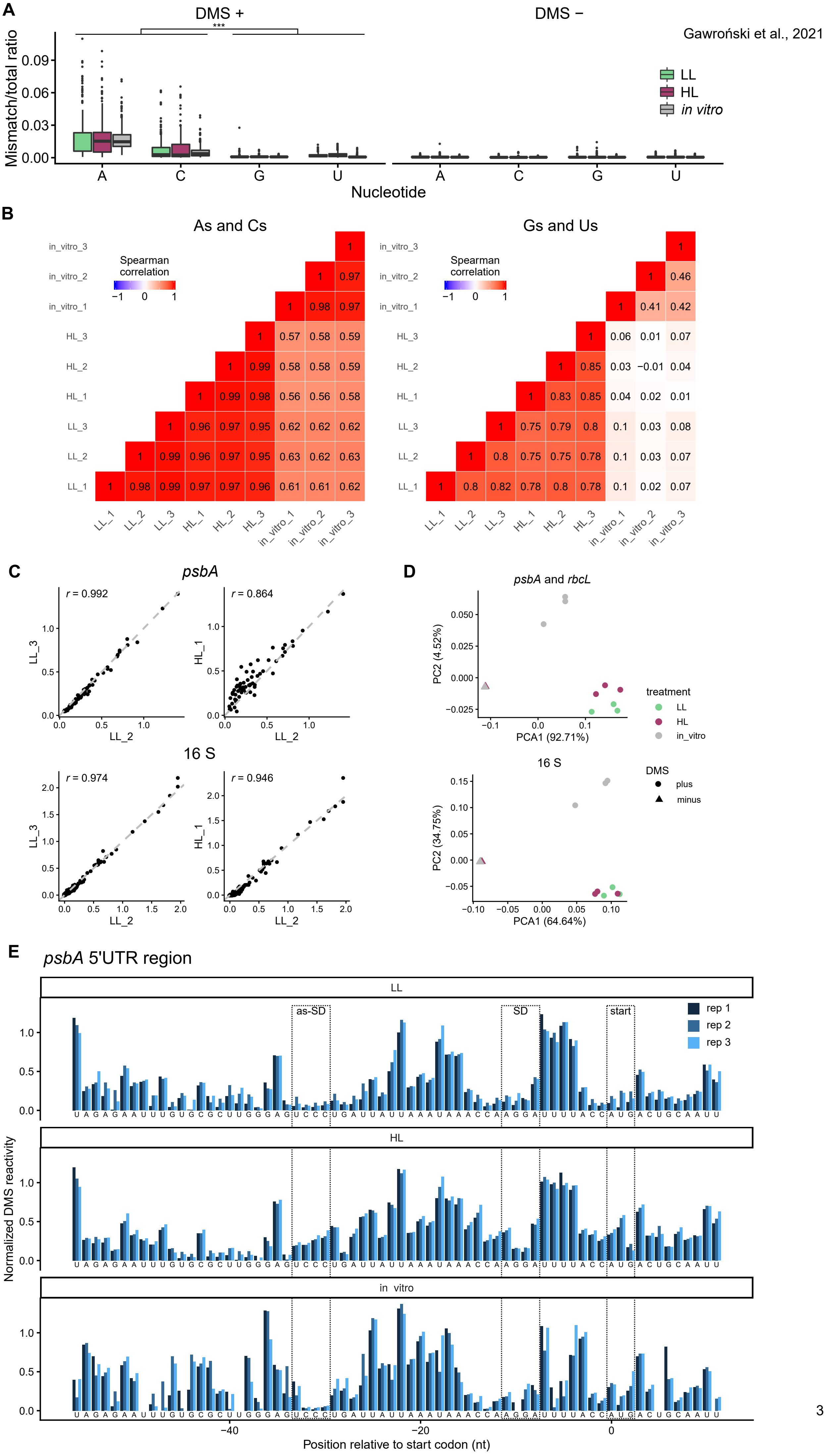
A
DMS +
DMS −
***
Gawroński et al., 2021
LL
HL
in vitro
Mismatch/total ratio
0.00
0.03
0.06
0.09
A
C
G
U
Nucleotide
B
As and Cs
Gs and Us
Spearman correlation
−1
0
1
in_vitro_3
in_vitro_2
in_vitro_1
HL_3
HL_2
HL_1
LL_3
LL_2
LL_1
C
psbA
r = 0.992
r = 0.864
LL_3
HL_1
LL_2
16 S
r = 0.974
r = 0.946
D
psbA and rbcL
PC2 (4.52%)
PCA1 (92.71%)
16 S
PC2 (34.75%)
PCA1 (64.64%)
treatment
LL
HL
in_vitro
DMS
plus
minus
E
psbA 5'UTR region
LL
HL
in_vitro
as-SD
SD
start
rep 1
rep 2
rep 3
Normalized DMS reactivity
U A G A G A A U U U G U G C G C U U G G G A G U C C C U G A U U A U U A A A U A A A C C A A G G A U U U U A C C A U G A C U G C A A U U
−40
−20
0
Position relative to start codon (nt)

Supplemental Figure S3 DMS-MaPseq quality control: A, Mismatch rates shown for each nucleotide as determined by MaPseq of DMS-probed samples (DMS +) and the water control (DMS −). Probed nucleotides are detected by mismatches between the known mRNA sequence and the sequence obtained by DMS-MaPseq. Adenosines and cytidines are statistically more significantly probed than guanosines and uridines. (*P*-values calculated with the Tukey HSD test; *** = $P < 0.001$). For the coverage see Supplemental Table 1. **B**, Spearman correlations between replicates and samples. For low light (LL), high light (HL), and *in vitro*-folded RNA samples three biological replicates each were included. The left part shows the values for adenosines and cytidines, the right part for guanosines und uridines. Spearman's *r* values are shown both numerically and as color codes (see scale). **C**, Pairwise comparisons of the probing of adenosines and cytidines of *psbA* translation initiation region (left) and 16S rRNA (right) of representative sample pairs. Spearman's *r* value is given. **D**, Principal component analysis (PCA) of the probing of adenosines and cytidines. The results for the 16S rRNA were analyzed separately from the ones for the two mRNAs (*psbA*, *rbcL*). Shown are the DMS-probed samples (DMS plus) and the water control (DMS minus). **E**, Normalized DMS reactivities for *psbA* in low light (LL), high light (HL), and *in vitro*-folded RNA samples with three biological replicates each. The positions of a sequence (as-SD) that can bind the Shine-Dalgarno sequence, the Shine-Dalgarno sequence (SD), and the start codon (start) are marked with dashed boxes (see Figure 1). The footprint of an RNA binding protein (Supplemental Figure S11A-D) is indicated in the sequence with a gray background.

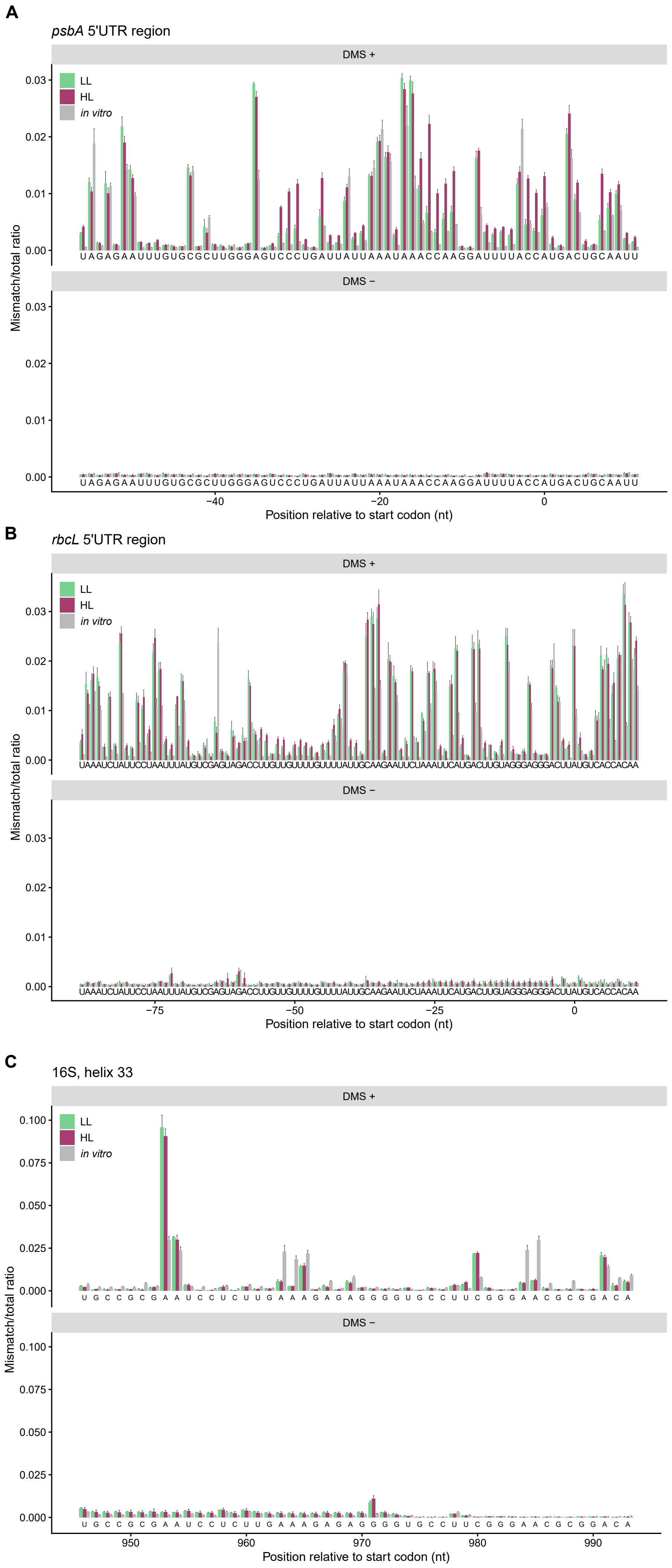

Supplemental Figure S4 DMS-MaPseq compared to water control: **A**, DMS-based mapping of

secondary structure of the *psbA* translation initiation region of 17-18-days-old plants (DMS +) compared to water control (DMS −) as determined by MaPseq. Control experiment for Figure 1A. The data for the low light (LL) control are shown in light green, for the high light (HL) sample in dark red, and for the mRNA that was allowed to fold *in vitro* in gray. **B**, Water control for DMS-MaPseq of the *rbcL* translation initiation region (Figure 3A). **C**, Water control for DMS-MaPseq of helix 33 of the 16S rRNA. Average values can be found in Supplemental Figure S3A.

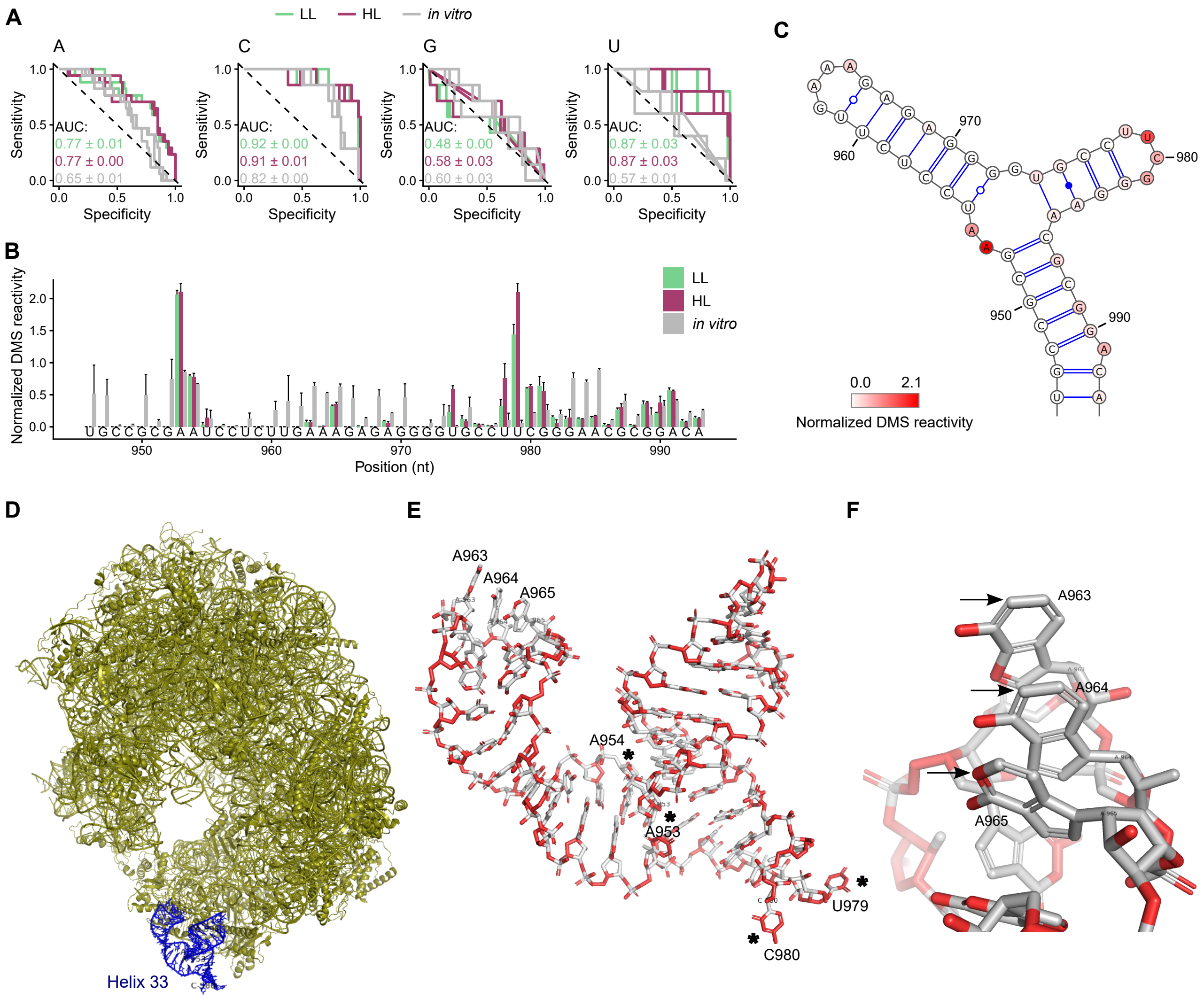

Supplemental Figure S5 DMS data reproduces known elements of the secondary structure of 16S rRNA: The secondary structure of helix 33 of the plastid 16S rRNA was analyzed by probing it with DMS. **A**, Receiver Operating Characteristics (ROC) curve generated using the Arabidopsis 16S rRNA secondary structure (Ahmed et al., 2017) as predictor and the normalized DMS reactivities as response. The ROC curves were calculated separately for each nucleotide (compare to Supplemental Figure S3A) and for each of the three biological replicates of each analyzed condition. Light green is the low light (LL) control, dark red the high light (HL) treatment, and gray RNA that was allowed to fold *in vitro*. From the generated ROC curve, the average area under curve (AUC) was calculated for each analyzed condition. As expected (Mustoe et al., 2019; Gawroński et al., 2020), the structure signal is better for adenosines and cytidines than for guanosines and uridines. In addition, the *in vivo* DMS

reactivities (LL, HL) reproduce the structure better than the ones at the *in vitro*-folded, protein-free 16S rRNA. **B**, Helix 33 was analyzed in more detail. High normalized DMS reactivity indicates high levels of bound probe, i.e. single-stranded regions. The *in-vivo* rRNA structure was not altered by the high light treatment, whereas there are multiple differences between the *in-vivo*, protein-bound 16S rRNA (LL and HL samples) and the *in-vitro*, protein-free 16S rRNA. **C**, The low-light data shown in **B** are here depicted as a simplified RNA structure based on the previously published structure of the plastid ribosome (Ahmed et al., 2017). DMS values for LL and HL samples fit the published ribosome structure well, with the exception of those for A963 and A964. **D**, Structure of the 16S rRNA (Ahmed et al., 2017), with helix 33 marked in blue. **E**, Magnification of helix 33 with adenosines A963-A965 as well (*) adenosines A953-A954, uridine U979, and cytidine C980 indicated. **F**, The accessibility of each atom in the loop was predicted as described in the Methods section; accessible atoms are marked in red. The atoms that should be susceptible to methylation by DMS are marked with arrows. The poor probing of A963 and A964 by DMS is not caused by their involvement in base pairing but by their (relative) inaccessibility to DMS.

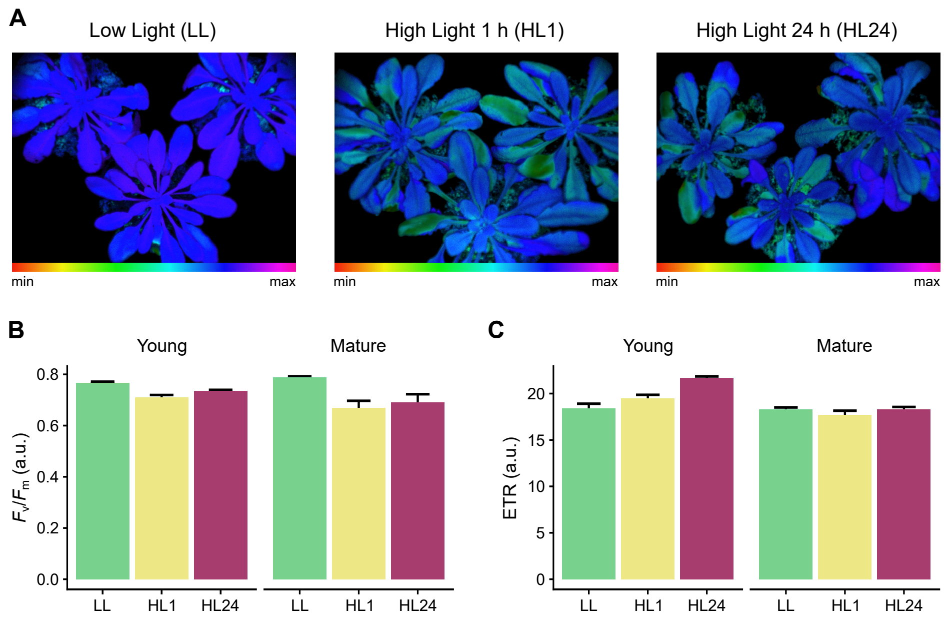

Supplemental Figure S6 Determination of photosynthetic parameters: **A**, False colored images of 7-week-old plants exposed to different light treatments showing the maximum quantum yields of photosystem II (F_v/F_m). Plants grown under control low light conditions (LL; 140-160 µE m^{-2} s^{-1}), after 1 h of high light (HL1; 1200 µE m^{-2} s^{-1}), and after 24 h of high light (HL24; 4 h, 1200 µE m^{-2} s^{-1}; 16 h dark; 4 h, 1200 µE m^{-2} s^{-1}) are compared. **B**, Average F_v/F_m values for young (not fully expanded) and mature (fully expanded) leaves of three plants for each condition shown in **A**. **C**, Average electron transport rate (ETR). Values are given in arbitrary units (a.u.). Error bars indicate mean standard errors.

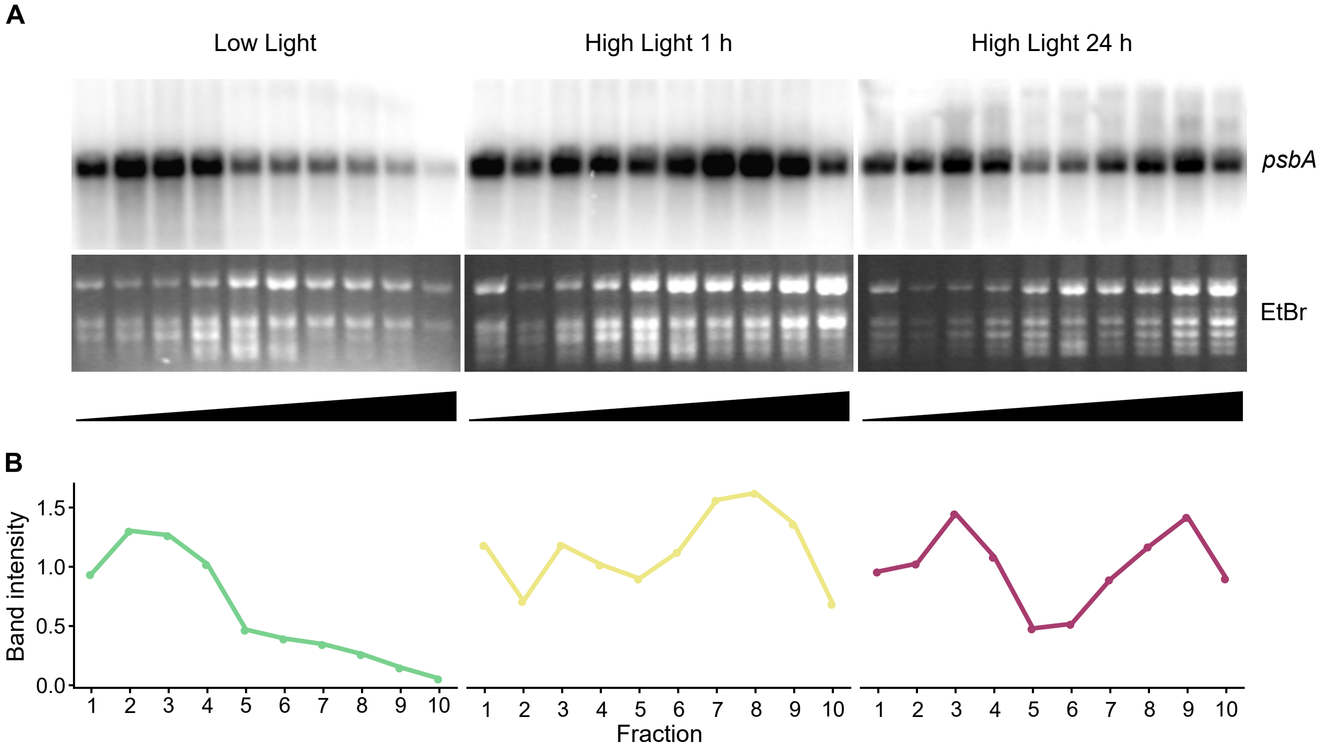

Supplemental Figure S7 Polysome analysis of *psbA* translation in mature plants: Comparison of low light control, 1 h high light and 24 h high light treatment (4 h high light, 16 h dark, 4 h high light). The low light control and the 24 h treatment were identical to the conditions used for the SHAPE experiment (Figure 1,3) and ribosome profiling (Figure 4). Plant extracts were subjected to sucrose-gradient centrifugation, fractionated into ten fractions, and RNA was extracted from each. The upper panels show RNA gel blots probed for *psbA* mRNA. The lower panels show ethidium bromide (EtBr)-stained rRNAs. The gradients are schematically depicted as black triangles at the bottom: less dense fractions containing free *psbA* mRNA are on the left, denser fractions containing polysome-associated *psbA* mRNA actively engaged in translation are on the right. **B**, Quantification of the amount of *psbA* mRNA normalized to the average amount of the five least dense fractions.

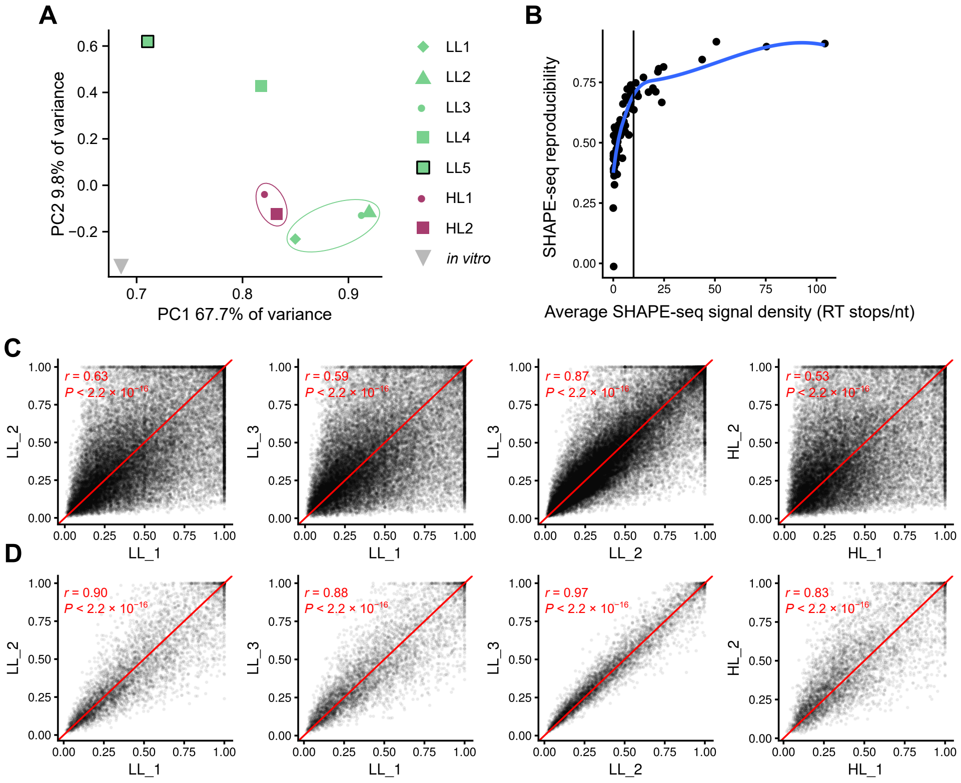

Supplemental Figure S8 Reproducibility of the mRNA secondary structure probing (NAI-N_3) data: **A**, Principal component analysis (PCA). The low light (LL) samples are shown in light green, the high light (HL) samples in dark red, and the sample that was folded *in vitro* is in gray. LL1 and LL2 are technical replicates separated after DNase treatment; the other samples are true biological replicates. Samples LL3, LL5, HL1, HL2, and the *in vitro* sample were depleted of rRNA prior to probing; the other samples are total RNA samples. Samples LL4 and LL5 were excluded from further analysis because of lack of similarity both to the other samples and to each other. The samples chosen for further analysis are encircled. **B**, Reproducibility is dependent on the average signal intensity (number of reverse transcription (RT) stops per nucleotide). The vertical line indicates the chosen cutoff of ≥10 reverse transcription stops/nt on average for each gene. Each point represents the value for one plastid-encoded gene. **C**, Pairwise comparisons of the three low light (LL1-LL3) and two high light samples (HL1, HL2). All data is represented, i.e. no cutoff is used. Each point represents the data for one nucleotide of a plastid mRNA. Spearman's *r* and *P* values are given. **D**, Pairwise comparisons of the same samples as in C but with a cutoff of ≥10 reverse transcription stops/nt on average for each gene.

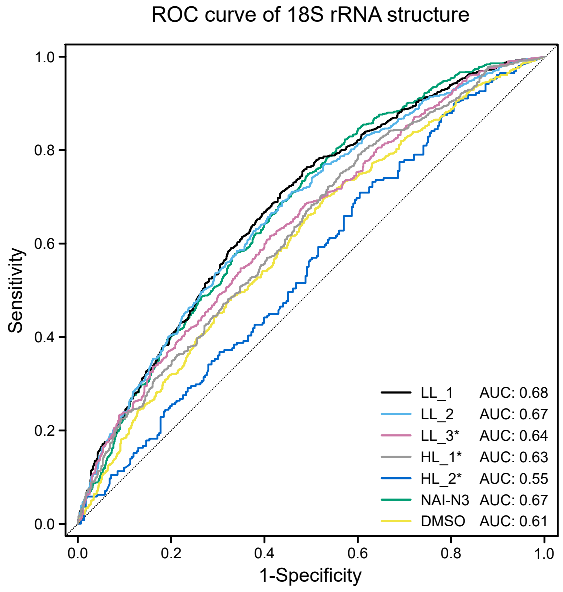

Supplemental Figure S9 Structural signal in SHAPE (NAI-N_3) data for the 18S rRNA structure: Receiver Operating Characteristics (ROC) curve generated using the Arabidopsis 18S rRNA secondary RNA structure from the CRW data as predictor (Cannone et al., 2002) and the swinsor-normalized termination counts as response. From the generated ROC curve, the area under curve (AUC) was calculated and indicated in the legend for each tested sample. Three low light samples (LL1-LL3) and two high light samples (HL1, HL2) are shown. A control without the biotin-based selection step (NAI-N_3) and a control treated only with DMSO are also included. The samples LL3, HL1, and HL2 (indicated with asterisks) were depleted of rRNA and had significantly lower coverage of the 18S rRNA, resulting in lower RNA structure probing signals.

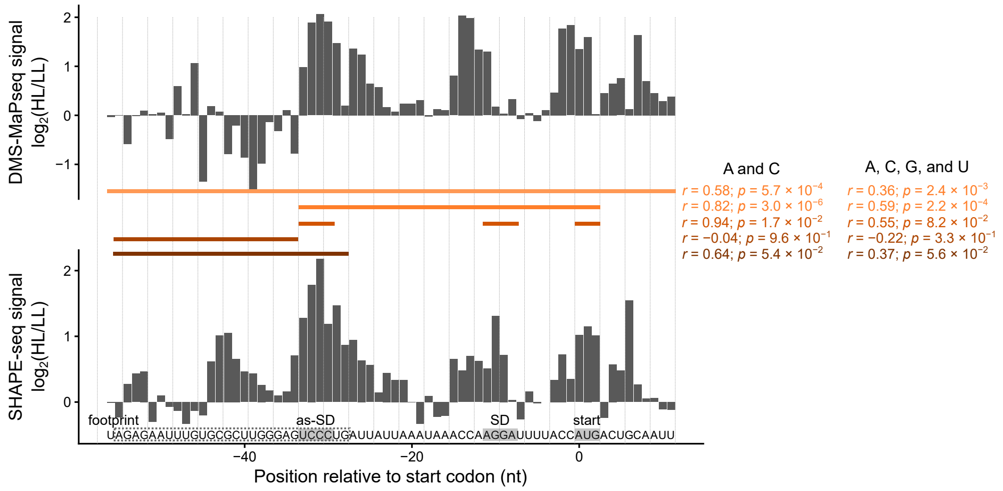

Supplemental Figure S10 Correlation between DMS and NAI-N_3 probing of the mRNA secondary structure of the *psbA* translation initiation region: Correlation analysis of SHAPE and DMS probing (Figure 1A,C). Shown are the ratios of the high light (HL) to the low light (LL) signals, expressed as $\log_2$ values. Spearman's r with the corresponding p values for the correlation of the SHAPE and DMS data for different parts of the *psbA* translation initiation region are given; the corresponding regions are indicated above the sequence in the same color. The correlation was analyzed separately for the more reliable probing of adenosines and cytidines (Supplemental Figures S3B, S5B) and for all four nucleotides. The structural data is well correlated at the *cis*-elements important for translation initiation but less well at the footprint (Figure S11A) excluding the sequence that can bind the SD (as-SD). This could be caused by differences between NAI-N_3 and DMS in the sensitivity to protein binding. DMS probing is sensitive to protein binding (Kwok et al., 2013; Talkish et al., 2014), whereas SHAPE reagents provide information about structural changes caused by protein binding, but bound proteins are not always detected as nucleotides with low reactivity (Spitale et al., 2013, 2015; Kenyon et al., 2015).

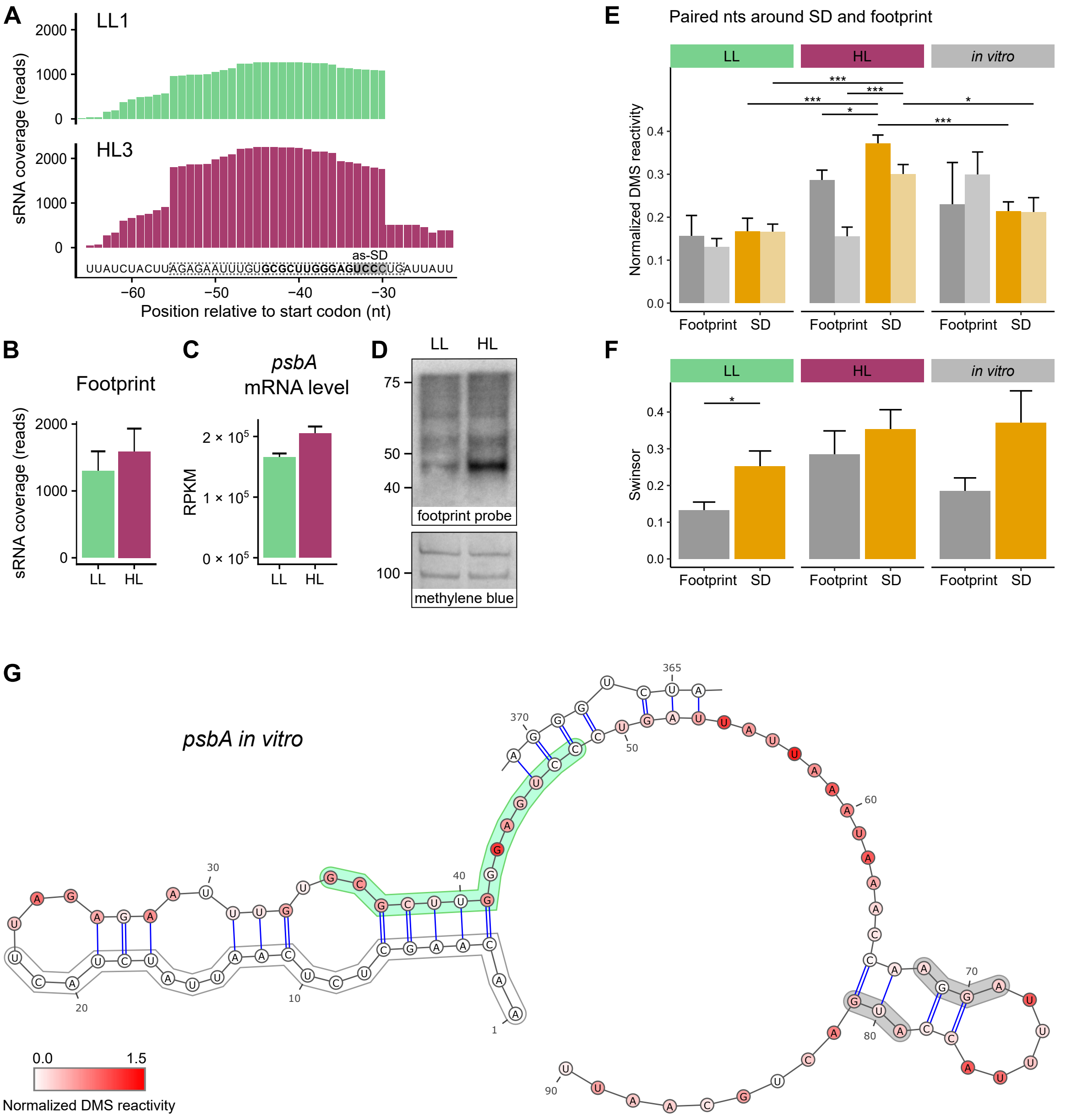

Supplemental Figure S11 Footprint of a putative regulatory protein bound to the 5' UTR of *psbA* and secondary structure of the *psbA* translation initiation region: **A**, The number of reads detected in the region of the footprint are shown for one example each of a low light control (LL1) and a high light treatment (HL3). MNase-digested RNA samples from 7-week-old plants were analyzed. The dashed box shows the position of the footprint found in this analysis, the bold nucleotides indicate the footprint of HCF173 (plus possibly other proteins) found earlier (McDermott et al., 2019), the sequence that can bind the Shine-Dalgarno sequence (as-SD) is marked in gray. **B**, Average coverage of the footprint in three biological replicates each for the low light control (light green) and high light samples (dark red). **C**, *psbA* mRNA levels in low light (light green) and after exposure to high light (dark red). Reads per kilobase of transcript per million mapped reads (RPKM) values are shown. **D**, Footprint detected in low light and high light samples by RNA gel blot analysis using a probe specific for the footprint. The same plant material as in **A** was used. The isolated RNA was not treated with any RNase.

Methylene blue-stained rRNAs are shown in the bottom panel as loading controls. Note that the footprint differs in size. It is shortest after RNase I treatment (McDermott et al., 2019) and longest when analyzed without any additional RNase treatment (**D**). **E**, Average normalized DMS reactivities of the nucleotides predicted to form base pairs in low light (Figure 2A) shown separately for the two halves of the stem loop: the footprint side (between nucleotides 35-48) and the SD side (between nucleotides 69-86) (compare Figure 2C). Values for low light (LL), high light (HL), and *in vitro*-folded RNA are shown. The darker colored columns represent the more reliable reactivities at adenosines/cytidines, the columns in lighter colors are the reactivities at all four nucleotides. Note also the sequence bias, especially the enrichment cytidines near the 3' end of the footprint (Figure 2A). Asterisks here (and in **F**) indicate statistically significant changes (*P*-values calculated with the Wilcoxon rank sum test; * = $P < 0.05$ and *** = $P < 0.001$), error bars indicate mean standard error. **F**, Average normalized SHAPE reactivities (swinsor) of the nucleotides predicted to form base pairs in low light (Figure 2A) shown separately for the two halves of the stem loop: the footprint side and the SD side. **G**, Predicted mRNA secondary structure of the *in vitro*-folded RNA using normalized DMS reactivities (Figure 1A) as constrains (control for Figure 2A,B). The prediction includes an interaction with a part of the coding region (position 364-371).

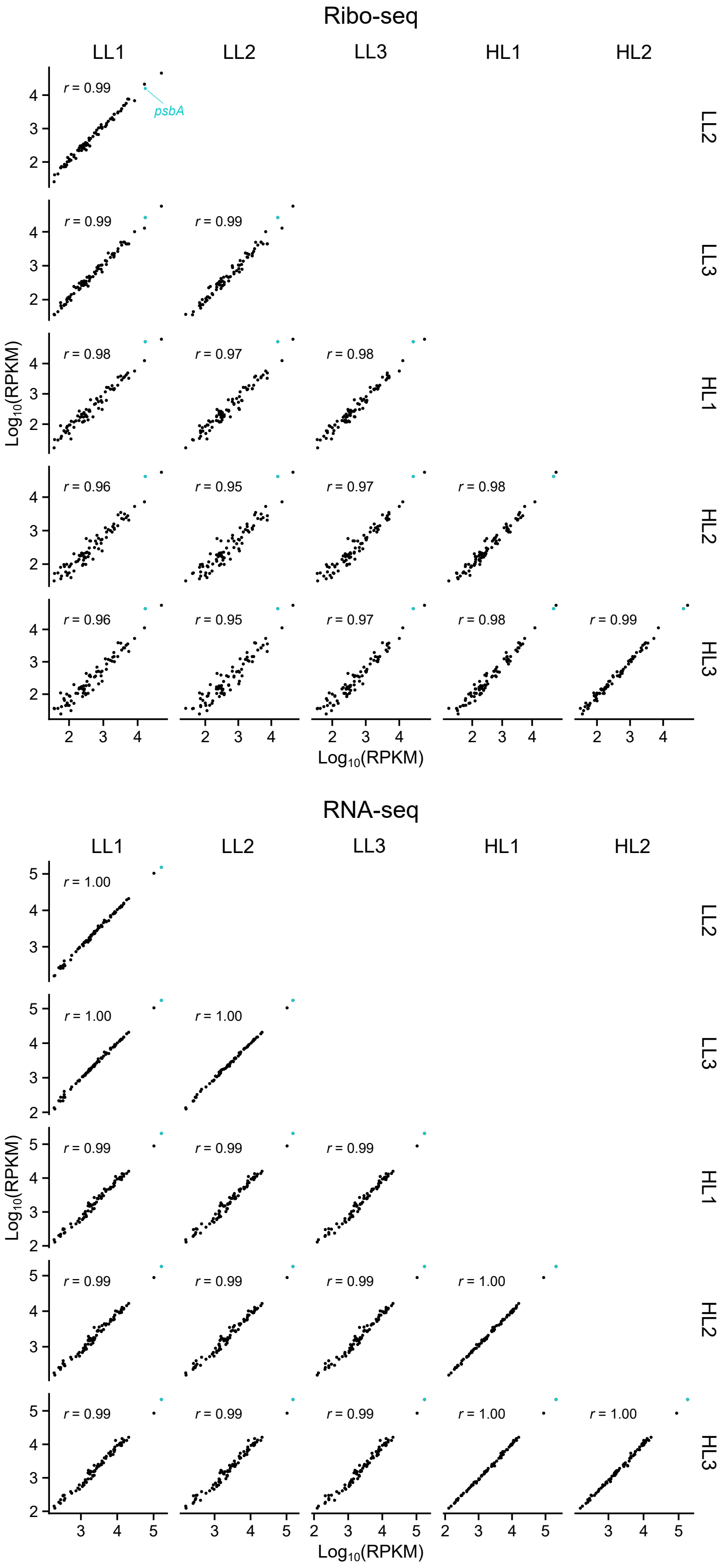

Supplemental Figure S12 Reproducibility of translatomic and transcriptomic data: Pairwise comparison of the plastid-encoded genes of the three replicates for both low light (LL) and high light (HL) (24 h). The upper part contains the translatomic (ribosome profiling data, Ribo-seq) data, the lower one the transcriptomic (RNA-seq) data. Each set of points represents the Log_{10} Reads per kilobase of transcript per million mapped reads (RPKM) values for one plastid-encoded coding region. Spearman's *r* value is given. *psbA* is marked in blue. The response to the high light treatment occurs primarily at the level of translation (see also Figure 4 and Supplemental Figure S13).

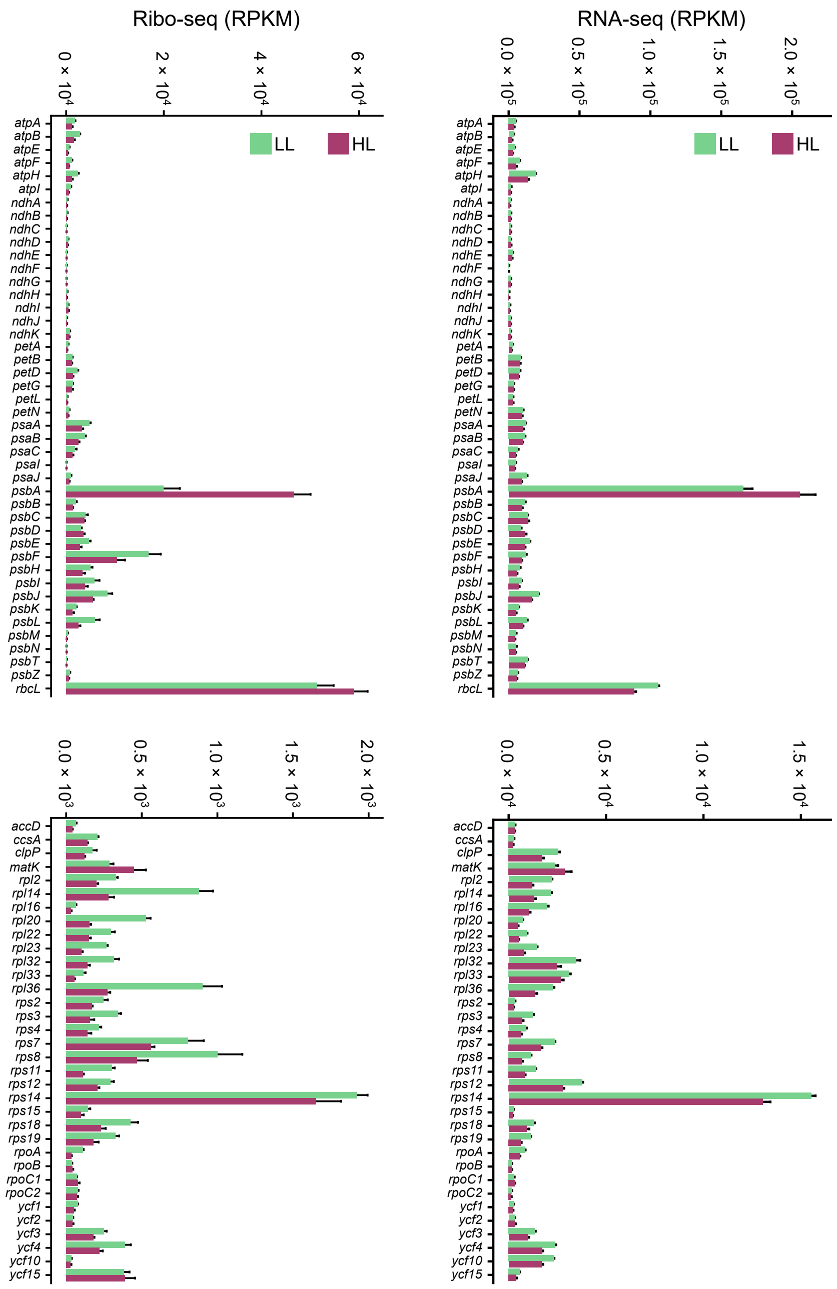

Supplemental Figure S13 Changes in translation and mRNA levels of plastid-encoded genes: Reads per kilobase of transcript per million mapped reads (RPKM) values are shown for each gene. The left panels depict the ribosome profiling data (Ribo-seq), the right panels show mRNA levels (RNA-seq). The values from the low light (LL) control are shown in light green, those from the 24h high light (HL) treatment in dark red. The data in the upper panels are derived from all genes coding for subunits of the photosynthetic complexes, the lower panels show the results for all other genes. Compare to the translation efficiency (Ribo-seq/RNA-seq) in Figure 4 (see also Figure S12 for data reproducibility).

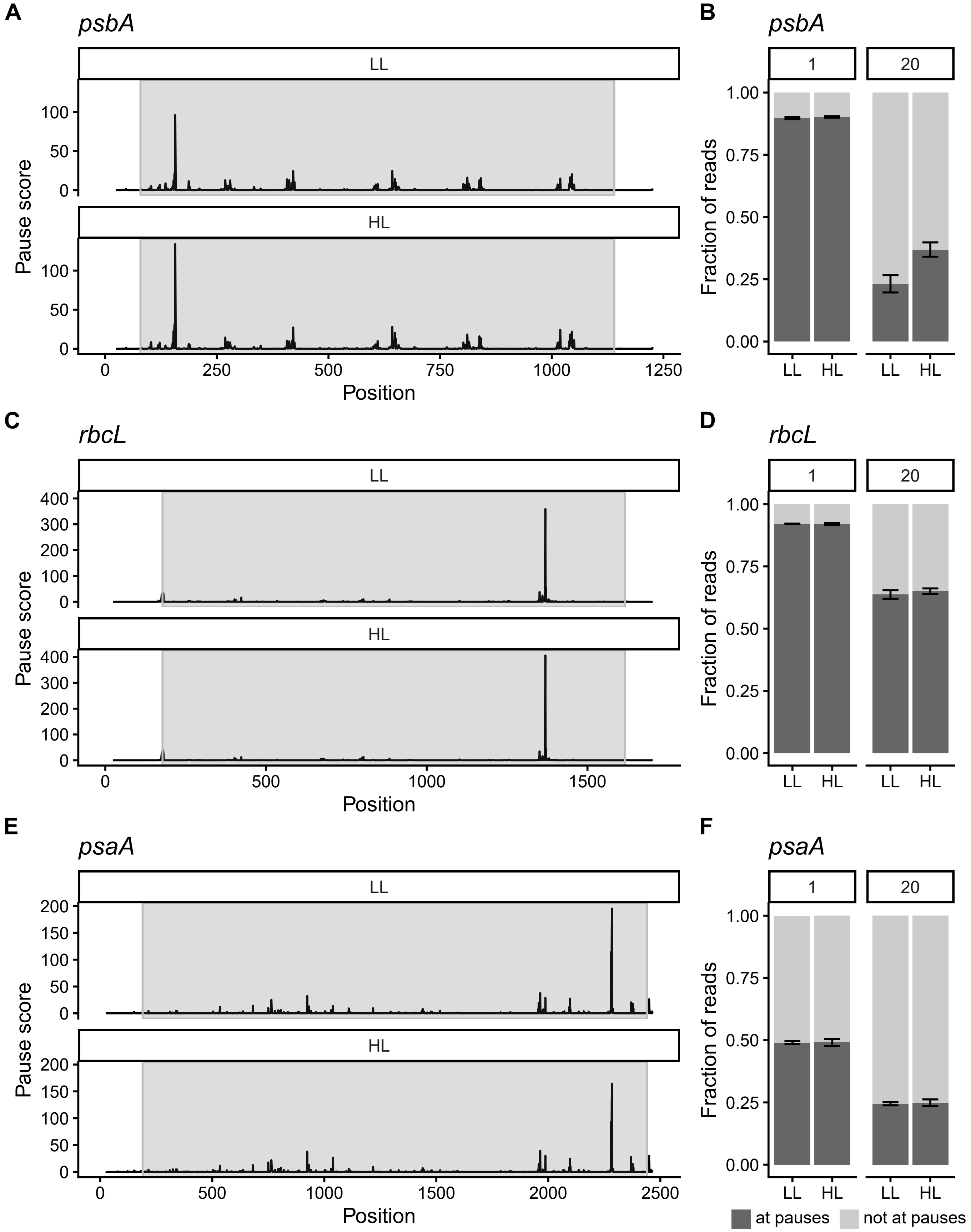

Supplemental Figure S14 Ribosome pausing on *psbA*, *rbcL*, and *psaA*: **A**, Ribosome profiling analysis of *psbA* using young leaves of 7-week-old plants (for change of translation see Figure 1G, 3H, and 4). The panel includes the position of the pause sites and their pause score (ribosome density at the pause site divided by the average ribosome density per nucleotide for the corresponding coding region). The

grey box indicates the coding region. Data analysis was done as described earlier (Gawroński et al., 2018). **B**, The fraction of the footprint reads assigned to pause sites at *psbA* is compared to the total number of reads. Pause sites are defined with two different cutoffs: a pause score of 1, i.e. all sites with a ribosome density above the average ribosome density, and 20, i.e. only pause sites with a 20 times higher ribosome density. Additional pause scores were tested, for the *psbA* fraction, the included values with a pause score of 20 showed the largest difference. The differences are not significant (Wilcoxon rank sum test). Moreover, the larger fraction of *psbA* reads assigned to sites with a pause score of 20 in high light compared to low light is insufficient to explain the increase in translation efficiency (Figure 1G). **C**, Position of pause sites and their pause score at *rbcL*. **D**, The fraction of footprint reads at pause sites at *rbcL*. **E**, Position of pause sites and their pause score at *psaA*. **F**, The fraction of footprint reads at pause sites at *psaA*.

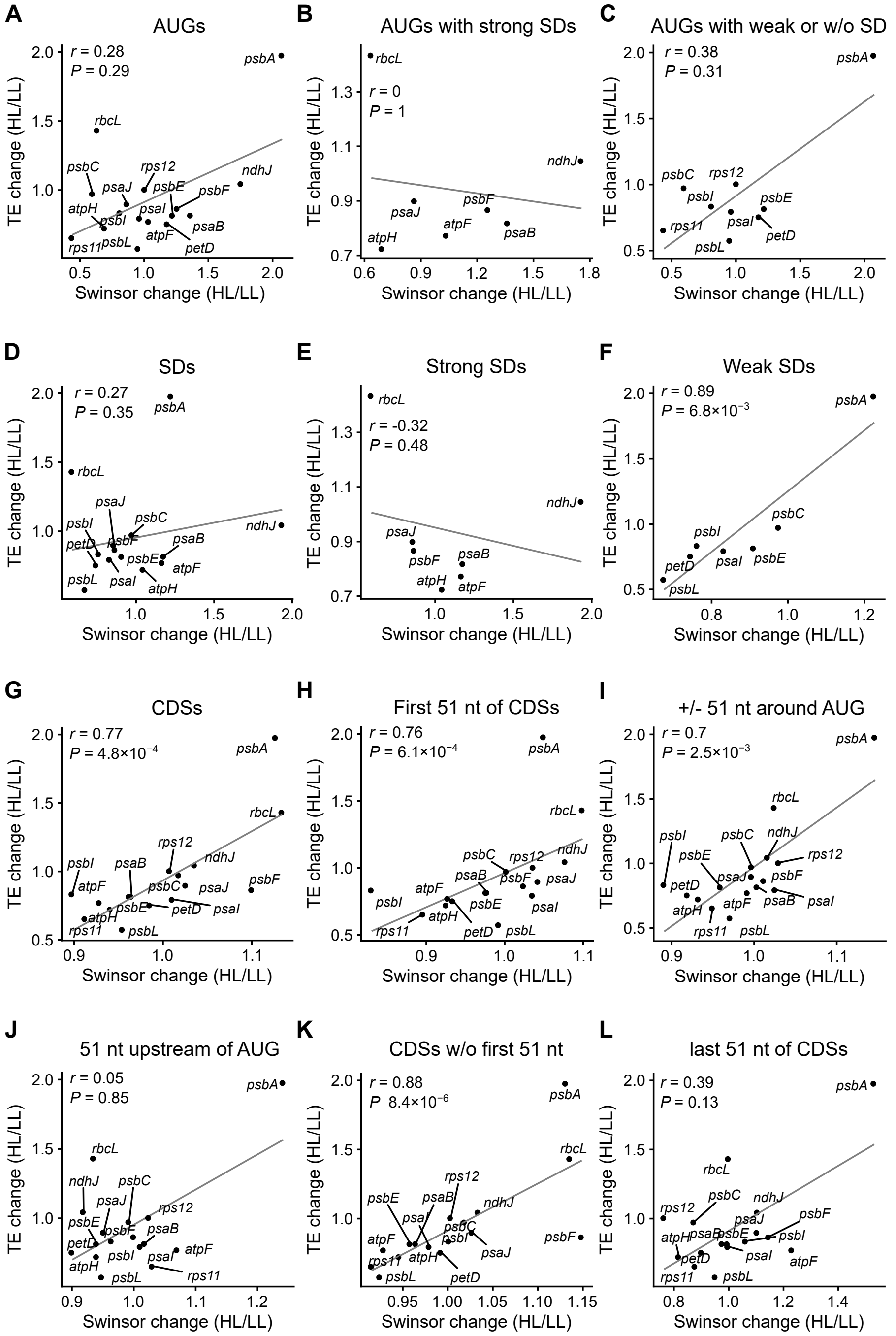

Supplemental Figure S15 Correlations between changes in mRNA secondary structure and translation efficiency: The changes in mRNA secondary structure are calculated from the swinsor-normalized termination count values derived from NAI-N_3 probing by dividing the values for the plants exposed to high light (HL) by those obtained from the low light (LL) controls. An increase in the swinsor value reflects a decrease in base pairing, i.e. less RNA secondary structure. Average changes for the indicated region of each gene are given. The change in translation efficiency is calculated by dividing the normalized read counts for the ribosomal footprints by the normalized read counts for the transcripts for each coding region, and then dividing the obtained values from the high light treatment by those from the low light control. Only genes with sufficient coverage of the mRNA secondary structure (in average at least 10 reverse transcription stops/nucleotide) are included. Spearman's *r* and *P* values are given. The correlation of the change of translation efficiency and the change of RNA

secondary structure at the following regions is shown: **A**, start codons (AUGs); **B**, start codons of genes with strong Shine-Dalgarno sequences (SD) (hybridization to the anti-SD of the 16S rRNA < −9 kcal mol^{-1}); **C**, start codons of genes with weak or without SD (> −6 kcal mol^{-1}); **D**, SDs; **E**, SDs of genes with strong SD (< −9 kcal mol^{-1}); **F**, SDs of genes with weak SD (> −6 kcal mol^{-1}); **G**, complete coding regions (CDSs); **H**, the first 51 nt of the coding regions including the start codons; **I**, the 51 nt around start codons; **J**, the 51 nt of the 5′ UTRs upstream of the start codons; **K**, coding regions without the first 51 nt, and **L**, the last 51 nt of the coding regions. We assume that, as described for *E. coli* (Mustoe et al., 2018), the correlation in the case of the CDS (G, K) is a consequence of differences in translation initiation, and that the structural changes of the CDS are caused by translating ribosomes reducing the mRNA secondary structure in the coding region.

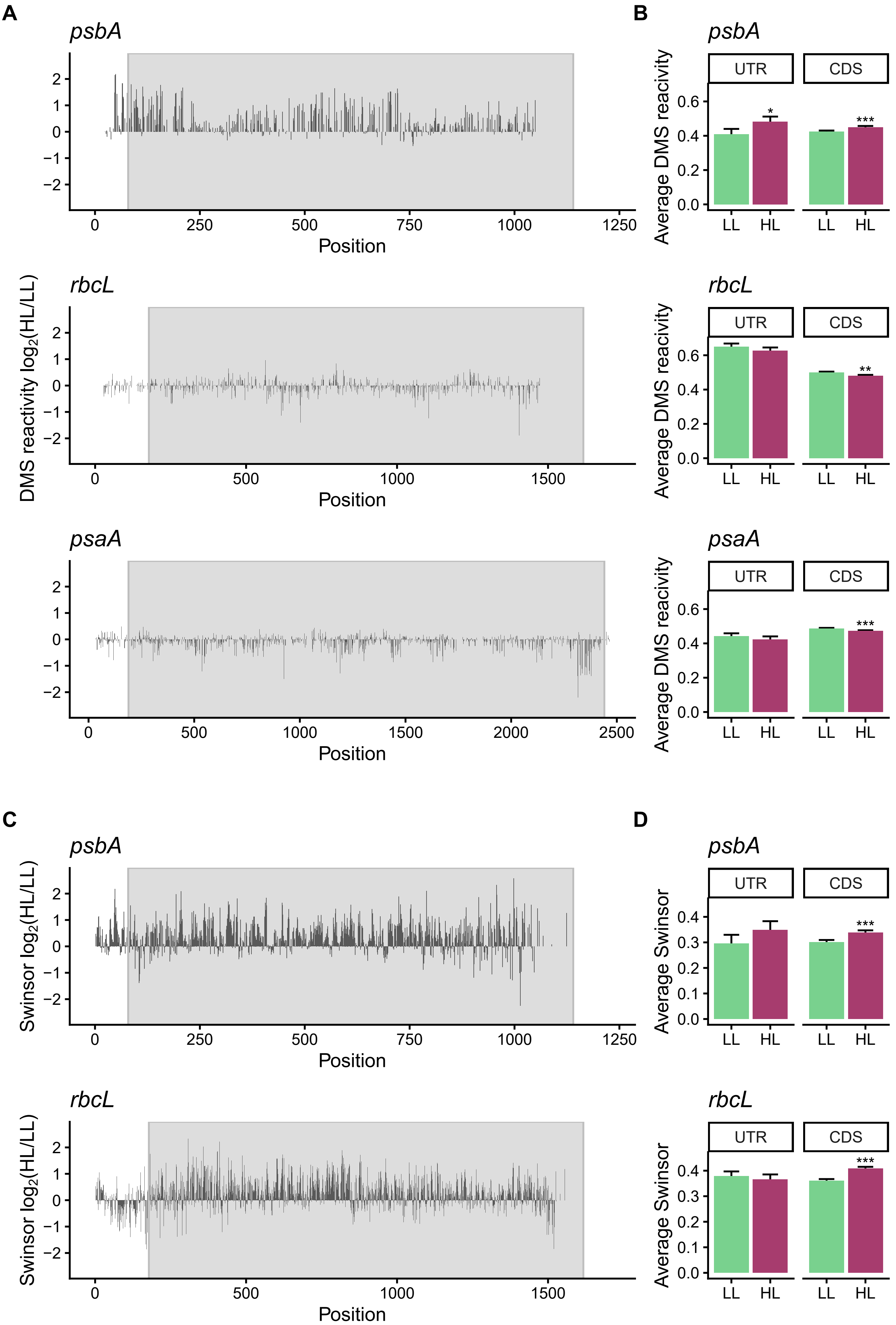

Supplemental Figure S16 mRNA secondary structure of the coding region of *psbA*, *rbcL*, and *psaA*:

A, DMS-MaPseq analysis using 17-18 days old plants (for change of translation see Figure 1B, 3G, and

S1) after low light (LL) and high light treatment (HL). The $\log_2$ of the HL/LL ratio of the DMS reactivities is included for a part of the 5’ UTR and the coding region (grey box). Probing and data analysis were done as described earlier (Gawroński et al., 2020). The low light values were published earlier (Gawroński et al., 2020). **B**, Average DMS reactivities for the 5’ UTR and coding region (CDS). *P*-values were calculated with the Wilcoxon rank sum test; * = $P < 0.05$, ** = $P < 0.01$, and *** = $P < 0.001$. The error bars depict the standard error of the mean. **C**, SHAPE analysis using young leaves of 7-week-old plants (for change of translation see Figure 1G, 3H, and 4). The $\log_2$ of the HL/LL ratio of the normalized SHAPE reactivity (swinsor) is shown. *psaA* is not included, because the coverage was not sufficient. **D**, Average swinsor values for the 5’ UTR and the coding region.

Supplemental Table 1 Number of mapped reads from the DMS-MaPseq analysis. Shown are the values for the three biological replicates for *in vitro* folded RNA, low light (LL), and high light (HL); both for the samples including DMS (DMS+) and the controls without DMS (DMS−).

**sample name**	***psbA***	**16S**	***rbcL***
in vitro 1 DMS+	59378	30168	11662
in vitro 2 DMS+	57866	25334	9423
in vitro 3 DMS+	68688	31449	14247
in vitro 1 DMS−	64056	43671	11989
in vitro 2 DMS−	60806	41152	13989
in vitro 3 DMS−	47834	31732	12155
LL 1 DMS+	51107	37692	16024
LL 2 DMS+	87031	39773	15312
LL 3 DMS+	57313	34316	15947
LL 1 DMS−	62580	31825	18332
LL 2 DMS−	52764	29991	16549
LL 3 DMS−	45224	27490	13855
HL 1 DMS+	73896	27997	14164
HL 2 DMS+	61158	37134	13469
HL 3 DMS+	67462	39944	17238
HL 1 DMS−	82393	31700	21708
HL 2 DMS−	56735	35606	15266
HL 3 DMS−	67588	39983	15526

Supplemental Table 2 Strength of binding of Shine-Dalgarno sequences to the anti-Shine-Dalgarno sequence: The strength of the hybridization of the nucleotides −22 to −2 of each 5′ UTR to the anti-Shine-Dalgarno sequence of the 16S rRNA was calculated *in silico* using Free2bind (Starmer et al., 2006) for all 16 genes whose RNA secondary structure could be analyzed. As comparison, the *psbA* and *rbcL* genes of *Nicotiana tabacum* and *Zea mays* are included. For each species, a typical growth temperature was used for the calculations (20 °C for Arabidopsis, 22 °C for *N. tabacum*, and 31 °C for *Z. mays*).

Species	Gene	SD-aSD binding [kcal/mol]	
Arabidopsis thaliana	*rbcL*	−12,98	strong SDs
	psaJ	−12,94	
	psaB	−12,84	
	psbF	−12,84	
	atpF	−9,74	
	atpH	−9,28	
	ndhJ	−9,24	
	psaI	−5,80	Weak SDs
	psbA	−5,50	
	psbE	−5,20	
	psbI	−5,20	
	petD	−4,84	
	psbL	−1,40	
	psbC	−0,84	
	rps11	1,14	No SD
	rps12	1,14	
Zea mays	*rbcL*	−11,23	
	psbA	1,51	
Nicotiana tabacum	*rbcL*	−12,66	
	psbA	1,21	

Supplemental Table 3 Fold change of mRNA levels and translation efficiency of nuclear-encoded genes encoding factors possibly regulating plastid translation: Log_2 values of the fold change in high light samples relative to the low light controls and the corresponding p values are given.

		mRNA levels		Translation efficiency	
Gene	Name	log_2 fold change	p value	log_2 fold change	p value
AT4G16390	ATP4, SVR7	−0,27	1,00E+00	−0,50	3,56E−01
AT1G77510	AtPDI6	0,16	1,00E+00	−0,16	7,51E−01
AT5G42310	CRP1	0,13	1,00E+00	−0,08	6,97E−01
AT3G46790	CRR2	0,60	1,00E+00	0,21	5,29E−01
AT3G17040	HCF107	0,04	1,00E+00	−0,15	6,10E−01
AT5G08720	HCF145	0,70	1,00E+00	−0,17	6,18E−01
AT3G09650	HCF152	1,04	1,00E+00	0,16	5,01E−01
AT1G16720	HCF173	0,03	1,00E+00	0,08	8,38E−01
AT4G35250	HCF244	0,50	1,00E+00	−0,35	1,57E−01
AT3G46610	LPE1	0,19	1,00E+00	−0,08	7,77E−01
AT4G34830	MRL1	0,45	1,00E+00	0,16	5,29E−01
AT5G02120	OHP1	0,28	1,00E+00	−0,22	3,83E−01
AT1G34000	OHP2	0,27	1,00E+00	−0,60	1,17E−01
AT1G71720	PBR1, PDE338, RLSB	−0,11	1,00E+00	0,20	6,18E−01
AT4G31850	PGR3	0,14	1,00E+00	0,15	5,03E−01
AT2G18940	PPR10	0,15	1,00E+00	−0,11	7,67E−01
AT5G46580	PPR53	0,42	1,00E+00	−0,14	7,66E−01

Supplemental Table 4 List of used oligonucleotides and their sequences. Blue are indexes.

Name	Sequence (5' --> 3')	Used for
psbAfor	AAGCGAAAGCCTATGGGGTC	probe for polysomes
psbArev	AATGTTGTGCTCAGCCTGGA	probe for polysomes
rbcLfor	GGACAACTGTGTGGACCGAT	probe for polysomes
rbcLrev	ATACCGCGGCTTCGATCTTT	probe for polysomes
psbA_footprint_probe	AGGGACTCCCAAGCGCACAAATTCTCT	probe for small RNA blot
DNA linker	CTGTAGGCACCATCAAT	determination of 3'-ends of plastid transcripts
Linker primer	5Phos/ATCTCGTATGCCGTCTTCTGCTTG/iSp18/CACTCA/iSp18/TCCGACGATCATTGATGGTGCCTACAG	determination of 3'-ends of plastid transcripts
RRN16S_1048_MaP_R1	CGGGACTTAACCCAACACC	RNA structure probing with DMS
psbA_MaP_RP	TGTAGATGGAGCCTCAACAGCA	RNA structure probing with DMS
rbcL_MaP_RP	AGATTGAGCCGAGTGCAATTAAACT	RNA structure probing with DMS
16S_540_MaP_IL_F	ACACGTTCAGAGTTCTACAGTCCGACGATCTTTAAGTCCGCCGTCAAATC	RNA structure probing with DMS
16S_1048_MaP_IL_R2	GAGTTCCTTGGCACCCGAGAATTCCACGGGACTTAACCCAACACC	RNA structure probing with DMS
psbA_1_MaP_ILMN_F	ACACGTTCAGAGTTCTACAGTCCGACGATCAACAAGCTCTCAATTATCTACT	RNA structure probing with DMS
psbA_174_MaP_ILMN_R2	GAGTTCCTTGGCACCCGAGAATTCCACCATCCAATGTAAAGACGGT	RNA structure probing with DMS
rbcL_1_MaP_IL_F	ACACGTTCAGAGTTCTACAGTCCGACGATCGTATTTGGCGAATCAAATATCATGGTCT	RNA structure probing with DMS
rbcL_578_MaP_IL_R	GAGTTCCTTGGCACCCGAGAATTCCACGTAGAGCAGCCAGGGCTTT	RNA structure probing with DMS
ILMN_PCR1	AATGATACGGCGACCACCGAGATCTACACGTTCAGAGTTCTACAGTCCGACGATC	RNA structure probing with DMS
ILMN_PCR2i1	CAAGCAGAAGACGGCATACGAGAT**CGTGAT**GTGACTGGAGTTCCTTGGCACCCGAGAATTCCA	RNA structure probing with DMS, in_vitro_1 DMS+
ILMN_PCR2i3	CAAGCAGAAGACGGCATACGAGAT**GCCTAA**GTGACTGGAGTTCCTTGGCACCCGAGAATTCCA	RNA structure probing with DMS, in_vitro_2 DMS+
ILMN_PCR2i8	CAAGCAGAAGACGGCATACGAGAT**TCAAGT**GTGACTGGAGTTCCTTGGCACCCGAGAATTCCA	RNA structure probing with DMS, in_vitro_3 DMS+
ILMN_PCR2i9	CAAGCAGAAGACGGCATACGAGAT**CTGATC**GTGACTGGAGTTCCTTGGCACCCGAGAATTCCA	RNA structure probing with DMS, in_vitro_1 DMS–
ILMN_PCR2i10	CAAGCAGAAGACGGCATACGAGAT**AAGCTA**GTGACTGGAGTTCCTTGGCACCCGAGAATTCCA	RNA structure probing with DMS, in_vitro_2 DMS–
ILMN_PCR2i11	CAAGCAGAAGACGGCATACGAGAT**GTAGCC**GTGACTGGAGTTCCTTGGCACCCGAGAATTCCA	RNA structure probing with DMS, in_vitro_3 DMS–
ILMN_PCR2i13	CAAGCAGAAGACGGCATACGAGAT**TTGACT**GTGACTGGAGTTCCTTGGCACCCGAGAATTCCA	RNA structure probing with DMS, LL_1 DMS+
ILMN_PCR2i14	CAAGCAGAAGACGGCATACGAGAT**GGAACT**GTGACTGGAGTTCCTTGGCACCCGAGAATTCCA	RNA structure probing with DMS, LL_2 DMS+
ILMN_PCR2i15	CAAGCAGAAGACGGCATACGAGAT**TGACAT**GTGACTGGAGTTCCTTGGCACCCGAGAATTCCA	RNA structure probing with DMS, LL_3 DMS+
ILMN_PCR2i16	CAAGCAGAAGACGGCATACGAGAT**GGACGG**GTGACTGGAGTTCCTTGGCACCCGAGAATTCCA	RNA structure probing with DMS, LL_1 DMS–
ILMN_PCR2i17	CAAGCAGAAGACGGCATACGAGAT**CTCTAC**GTGACTGGAGTTCCTTGGCACCCGAGAATTCCA	RNA structure probing with DMS, LL_2 DMS–
ILMN_PCR2i18	CAAGCAGAAGACGGCATACGAGAT**GCGGAC**GTGACTGGAGTTCCTTGGCACCCGAGAATTCCA	RNA structure probing with DMS, LL_3 DMS–
ILMN_PCR2i19	CAAGCAGAAGACGGCATACGAGAT**TTTCAC**GTGACTGGAGTTCCTTGGCACCCGAGAATTCCA	RNA structure probing with DMS, HL_1 DMS+
ILMN_PCR2i20	CAAGCAGAAGACGGCATACGAGAT**GGCCAC**GTGACTGGAGTTCCTTGGCACCCGAGAATTCCA	RNA structure probing with DMS, HL_2 DMS+
ILMN_PCR2i21	CAAGCAGAAGACGGCATACGAGAT**CGAAAC**GTGACTGGAGTTCCTTGGCACCCGAGAATTCCA	RNA structure probing with DMS, HL_3 DMS+
ILMN_PCR2i22	CAAGCAGAAGACGGCATACGAGAT**CGTACG**GTGACTGGAGTTCCTTGGCACCCGAGAATTCCA	RNA structure probing with DMS, HL_1 DMS–
ILMN_PCR2i23	CAAGCAGAAGACGGCATACGAGAT**CCACTC**GTGACTGGAGTTCCTTGGCACCCGAGAATTCCA	RNA structure probing with DMS, HL_2 DMS–
ILMN_PCR2i24	CAAGCAGAAGACGGCATACGAGAT**GCTACC**GTGACTGGAGTTCCTTGGCACCCGAGAATTCCA	RNA structure probing with DMS, HL_3 DMS–
RT_15xN	AGACGTGTGCTCTTCCGATCTNNNNNNNNNNNNNNN	RNA structure probing with NAI-N3
Ligation_adapter	PHO-NNNNNNNAGATCGGAAGAGCGTCGTGTAGGGAAAGAGTGT-3NHC3	RNA structure probing with NAI-N3
PCR_forward primer	AATGATACGGCGACCACCGAGATCTACACTCTTTCCCTACACGACGCT	RNA structure probing with NAI-N3
PCR_reverse_index.2	CAAGCAGAAGACGGCATACGAGAT**ACATCG**GTGACTGGAGTTCAGACGTGTGCTCTTCCGATCT	RNA structure probing with NAI-N3, sample LL1
PCR_reverse_index.4	CAAGCAGAAGACGGCATACGAGAT**TGGTCA**GTGACTGGAGTTCAGACGTGTGCTCTTCCGATCT	RNA structure probing with NAI-N3, sample LL4
PCR_reverse_index.5	CAAGCAGAAGACGGCATACGAGAT**CACTGT**GTGACTGGAGTTCAGACGTGTGCTCTTCCGATCT	RNA structure probing with NAI-N3, sample LL3
PCR_reverse_index.8	CAAGCAGAAGACGGCATACGAGAT**TCAAGT**GTGACTGGAGTTCAGACGTGTGCTCTTCCGATCT	RNA structure probing with NAI-N3, sample NAI-N3
PCR_reverse_index.9	CAAGCAGAAGACGGCATACGAGAT**CTGATC**GTGACTGGAGTTCAGACGTGTGCTCTTCCGATCT	RNA structure probing with NAI-N3, sample DMSO
PCR_reverse_index10	CAAGCAGAAGACGGCATACGAGAT**AAGCTA**GTGACTGGAGTTCAGACGTGTGCTCTTCCGATCT	RNA structure probing with NAI-N3, sample in vitro
PCR_reverse_index12	CAAGCAGAAGACGGCATACGAGAT**TACAAG**GTGACTGGAGTTCAGACGTGTGCTCTTCCGATCT	RNA structure probing with NAI-N3, sample LL5
PCR_reverse_index13	CAAGCAGAAGACGGCATACGAGAT**TTGACT**GTGACTGGAGTTCAGACGTGTGCTCTTCCGATCT	RNA structure probing with NAI-N3, sample LL3
PCR_reverse_index14	CAAGCAGAAGACGGCATACGAGAT**GGAACT**GTGACTGGAGTTCAGACGTGTGCTCTTCCGATCT	RNA structure probing with NAI-N3, sample HL1
PCR_reverse_index15	CAAGCAGAAGACGGCATACGAGAT**TGACAT**GTGACTGGAGTTCAGACGTGTGCTCTTCCGATCT	RNA structure probing with NAI-N3, sample HL2

Supplemental References

Ahmed, T., Shi, J., and Bhushan, S. (2017). Unique localization of the plastid-specific ribosomal proteins in the chloroplast ribosome small subunit provides mechanistic insights into the chloroplastic translation. Nucleic Acids Res. **45**: 8581–8595.

Cannone, J.J. et al. (2002). The comparative RNA web (CRW) site: an online database of comparative sequence and structure information for ribosomal, intron, and other RNAs. BMC Bioinformatics **3**: 2.

Gawroński, P., Jensen, P.E., Karpiński, S., Leister, D., and Scharff, L.B. (2018). Pausing of Chloroplast Ribosomes Is Induced by Multiple Features and Is Linked to the Assembly of Photosynthetic Complexes. Plant Physiol. **176**: 2557–2569.

Gawroński, P., Pałac, A., and Scharff, L.B. (2020). Secondary Structure of Chloroplast mRNAs In Vivo and In Vitro. Plants (Basel, Switzerland) **9**: 323.

Kenyon, J.C., Prestwood, L.J., and Lever, A.M.L. (2015). A novel combined RNA-protein interaction analysis distinguishes HIV-1 Gag protein binding sites from structural change in the viral RNA leader. Sci. Rep. **5**: 14369.

Kwok, C.K., Ding, Y., Tang, Y., Assmann, S.M., and Bevilacqua, P.C. (2013). Determination of in vivo RNA structure in low-abundance transcripts. Nat. Commun. **4**: 2971.

McDermott, J.J., Watkins, K.P., Williams-Carrier, R., and Barkan, A. (2019). Ribonucleoprotein Capture by in Vivo Expression of a Designer Pentatricopeptide Repeat Protein in Arabidopsis. Plant Cell **31**: 1723–1733.

Mustoe, A.M., Busan, S., Rice, G.M., Hajdin, C.E., Peterson, B.K., Ruda, V.M., Kubica, N., Nutiu, R., Baryza, J.L., and Weeks, K.M. (2018). Pervasive Regulatory Functions of mRNA Structure Revealed by High-Resolution SHAPE Probing. Cell **173**: 181-195.e18.

Mustoe, A.M., Lama, N.N., Irving, P.S., Olson, S.W., and Weeks, K.M. (2019). RNA base-pairing complexity in living cells visualized by correlated chemical probing. Proc. Natl. Acad. Sci. U. S. A. **116**: 24574–24582.

Spitale, R.C., Crisalli, P., Flynn, R. a, Torre, E.A., Kool, E.T., and Chang, H.Y. (2013). RNA SHAPE analysis in living cells. Nat. Chem. Biol. **9**: 18–20.

Spitale, R.C., Flynn, R.A., Zhang, Q.C., Crisalli, P., Lee, B., Jung, J.-W., Kuchelmeister, H.Y., Batista,

P.J., Torre, E.A., Kool, E.T., and Chang, H.Y. (2015). Structural imprints in vivo decode RNA regulatory mechanisms. Nature **519**: 486–90.

Starmer, J., Stomp, A., Vouk, M., and Bitzer, D. (2006). Predicting Shine-Dalgarno sequence locations exposes genome annotation errors. PLoS Comput. Biol. **2**: e57.

Talkish, J., May, G., Lin, Y., Woolford, J.L., and McManus, C.J. (2014). Mod-seq: high-throughput sequencing for chemical probing of RNA structure. RNA **20**: 713–20.
